# Protocol for a Single-Arm Feasibility Study of Photobiomodulation for Fatigue, Depression, and Pain in Inflammatory Bowel Disease

**DOI:** 10.3390/biomedicines11082179

**Published:** 2023-08-02

**Authors:** Tatjana Ewais, Jakob Begun, E-Liisa Laakso

**Affiliations:** 1 School of Medicine, University of Queensland, St Lucia, QLD 4068, Australia; jakob.begun@mater.uq.edu.au; 2 Mater Adolescent and Young Adult Health Clinic, Mater Misericordiae Ltd., South Brisbane, QLD 4101, Australia; 3 School of Medicine and Dentistry, Gold Coast Campus, Griffith University, Southport, QLD 4215, Australia; 4 Mater Research Institute, University of Queensland, South Brisbane, QLD 4101, Australia; liisa.laakso@mater.uq.edu.au; 5 Menzies Health Institute Queensland, Gold Coast Campus, Griffith University, Southport, QLD 4215, Australia

**Keywords:** photobiomodulation, fatigue, depression, pain, IBD, youth

## Abstract

Background: There are limited treatment options for mental health comorbidities associated with Inflammatory Bowel Disease (IBD), although they have been shown to negatively affect the course of IBD and multiple important areas of functioning. Photobiomodulation (PBM) is a new therapeutic intervention using laser-generated low-powered light therapy that has shown early promise in alleviating fatigue, depression, and pain in chronic illness. Methods: This prospective, single-arm pilot study aims to assess the feasibility and efficacy of PBM in the treatment of fatigue, depression, and pain in youth with IBD. We will recruit 28 young adults with IBD who will receive PBM in addition to treatment as usual. The primary outcome will be fatigue, while secondary outcomes will include depression, pain, quality of life, inflammatory markers, alterations in microbiome composition, physical activity, and functioning. Outcome measures will be assessed at baseline, after a 10-week control period (pre-PBM), at 20 weeks (post-PBM), and at 30 weeks. Feasibility will be assessed by attendance, recruitment rates, and participants’ views of PBM. Mixed-effects linear regression modelling will be used to assess the PBM effect on continuous outcomes (fatigue, depression, anxiety and stress scores, and inflammation levels). Results: The study will provide preliminary indicators of PBM feasibility and efficacy in IBD.

## 1. Introduction

Inflammatory Bowel Disease (IBD) is an immune-mediated disease characterised by chronic inflammation of the gastrointestinal tract, and high levels of fatigue, depression, pain and other extraintestinal manifestations that worsen the course of IBD, impair quality of life and contribute to high burden of disease [1,2,3,4].

Fatigue prevalence rates range between 40–48% in remission, and up to 86% in moderate-to-severely active IBD [5]. The aetiology and persistence of IBD-related fatigue is likely to be multifactorial and associated with complex immune mechanisms including genetics, epigenetics, and environmental and drug treatment-related effects. Specifically, these factors include continuing low-grade systemic inflammation, IBD medication side effects, nutritional deficiencies, comorbid depression, and other comorbidities, such as joint pain, respiratory and cardiovascular disease, and poor sleep quality [6]. There is no known effective method of treating IBD-related fatigue [5,6].

Fatigue is likely mediated by both the central nervous system (CNS) and peripheral neuromuscular mechanisms [6]. In chronic disease, fatigue is associated with unrefreshing sleep, reduced physical functioning, and depression and is also part of depressive symptoms [6].

Depression rates in individuals with IBD during periods of remission are 2–3 times higher than in the general population, ranging between 24 and 27%, and several times higher during exacerbations with reported rates of depression during flares of over 60% [7]. Young adults with IBD have significantly higher rates of depression than their healthy peers and young people with other chronic conditions. Given the bidirectional relationship between depression and IBD [8], treatment of depression is likely to improve IBD symptoms and its prognosis. Since depression is strongly associated with the presence and severity of IBD-related fatigue and fatigue is a common symptom of depression [9], treating depression can improve fatigue and vice versa, and treatment of both can improve quality of life and IBD course. The impact of depression on fatigue is likely to be twofold as depression can both cause and worsen fatigue through reduced energy levels and anhedonia (the inability to feel pleasure in normally pleasurable activities), which are symptoms of depression, and through its impact on the IBD course [3,4,9]. 

Depression has been shown to be associated with IBD-related pain with multiple postulated mechanisms of their co-occurrence and interaction, including common underlying inflammatory pathways, amplification of pain signals by depression and stress and reduced adherence to medications [9,10]. One of the most common presenting symptoms of Crohn’s disease is abdominal pain, and one of the most frequently reported extra-intestinal symptoms of IBD is the pain of seronegative spondyloarthropathy affecting the large joints [11]. 

Since fatigue and depression in the setting of IBD are associated with an increase in IBD-related pain [10], successful treatments are likely to work through addressing their common underlying causes. However, there is no single effective treatment for these inter-related symptoms of IBD, and those which are used are most likely to be biologics or pharmacologically based, thus with the potential for side-effects, loss of efficacy or resistance. Photobiomodulation is, therefore, a promising novel alternative treatment option which may augment the treatment of IBD-related fatigue, depression, and pain.

## 2. Photobiomodulation in Treatment of Fatigue, Depression, and Pain

Photobiomodulation is the use of photonic (light) energy to regulate and enhance cellular functioning. The effect of PBM is thought to occur through the interactions of photons with the cytochrome c complex in mitochondria which leads to improved cellular metabolism and tissue regeneration. When used for pre-conditioning before exercise, PBM has been shown to have moderate evidence for improving physical performance [12,13] and low to moderate evidence for reducing muscle fatigue [13,14].

Additional beneficial effects of PBM on physical performance include reduced muscle fatigue in a variety of settings and conditions [15], most likely by increasing local matching of bulk and microvascular oxygen delivery relative to muscle oxygen utilisation [16]. 

The evidence for the use of PBM in the treatment of depression is emerging, with recent reviews identifying PBM as a promising novel treatment that has been well tolerated by people with depression [17,18]. In Parkinson’s disease, PBM has been shown to improve sleep-wake cycles [19] critical in improving fatigue symptoms. 

The effectiveness of PBM in managing pain has been well supported by research [20,21,22]. Author E-LL has researched the effect of different wavelengths and doses of PBM in people with chronic pain and described the link between the peripheral application of PBM and central nervous system effects and analgesic mechanisms of PBM [23].

There is emerging evidence on the positive effect of PBM on the gut microbiome. Liebert et al. [24] have investigated PBM in a mouse model and demonstrated that PBM (red and infrared wavelengths) can alter microbiome diversity in healthy mice and increase numbers of *Allobaculum*, a bacterium associated with a healthy microbiome. Zanotta and colleagues [25] applied PBM to consenting participants with a history of oral mucositis following cancer therapies. They found that PBM modulated the inflammatory response by reducing the proinflammatory cytokines and changed the microbiome composition with increased diversity and an increase in the number of known beneficial bacteria. 

Taken together, these research results suggest that PBM can improve fatigue, depression, pain, and microbiome diversity in chronic disease. Despite this, there have been no trials or reviews of PBM in IBD to date apart from our group’s recent article on the mechanism of PBM in IBD [26]. This prospective, single-arm pilot study of PBM in youth with IBD will therefore assess the feasibility and preliminary efficacy of PBM in IBD. Should improvement in fatigue markers and/or depressive symptoms be found, the research will progress to a larger randomised, placebo-controlled study.

## 3. Study Goals, Objectives, and Hypotheses

The goal of this research is to determine the feasibility of progressing to a larger study regarding the possible effect of PBM for managing the symptoms of fatigue, depression, and pain experienced by young adults with IBD. 

The primary objective is to determine if the protocol and the intervention are acceptable to participants, and feasible to deliver. Acceptability/tolerability/feasibility of the intervention will be assessed quantitatively, through measures of attendance/attrition/drop-out and qualitatively, through assessing participants’ experiences and views of the intervention through the post-PBM evaluation questionnaire. In conjunction, we wish to understand if PBM (delivered per protocol) influences fatigue (measured using the FACIT-Fatigue questionnaire). The FACIT-Fatigue questionnaire has been validated for use in IBD [27].

The secondary objectives are to evaluate the effect of PBM on depression (measured using the short form of the Depression, Anxiety and Stress Scale DASS-21) and health-related quality of life (measured by the Short Form 36; SF-36); levels of inflammation (Erythrocyte Sedimentation Rate-ESR, C Reactive Protein-CRP and Faecal Calprotectin-FCP); the gut microbiome (by quantity and type of bacterial phyla and genera found in stool samples); abdominal and joint pain, physical activity (measured by the International Physical Activity Questionnaire-IPAQ), and physical function (Patient-specific Functional Scale). We will use data from this study to inform the design and sample size calculation for a future RCT. 

### Hypotheses

**Hypothesis** **1.** *PBM intervention will be feasible and acceptable*.

**Hypothesis** **2.** *PBM intervention will result in significantly improved fatigue scores post-intervention*.

**Hypothesis** **3.** *PBM will decrease depression and increase QoL*.

**Hypothesis** **4.** *PBM will result in decreased abdominal and joint pain, increased physical activity, and physical function*.

**Hypothesis** **5.** *PBM intervention will be associated with decreased inflammatory burden*.

**Hypothesis** **6.** *PBM intervention will be associated with an increase in the alpha diversity of the gut microbiome with an increase in the number of known beneficial bacteria and decrease in number of potentially pathogenic genera*.

## 4. Materials and Methods

This is a prospective, single-arm pilot study. The study is designed as a pre–post study with three phases, each with a duration of ten weeks, and four data collection points. The three study phases include a 10-week observation period, followed by a 10-week PBM intervention phase during which the participants will receive weekly PBM, and the study concludes with a second 10-week observation or washout phase. Data collection points include baseline, pre-PBM, post-PBM, and the final collection point, 10 weeks after the completion of the PBM intervention. The purpose of the post-intervention observational period is to record and evaluate any sustained effects of PBM. The study duration is expected to be up to 12 months, from commencement of recruitment to end of data collection of the final participant. Rolling recruitment will be used until the required sample of 28 is achieved. 

The primary outcome of interest will be fatigue while secondary outcomes will include depression, pain, quality of life, inflammatory markers, and microbiome characterisation (of phyla and genera), physical activity, and physical functioning. All outcome measures, other than pain, will be assessed at the 4 data collection points. Pain will be assessed weekly as evidence shows that this frequency will provide most accurate assessment. 

Feasibility of the study procedures and PBM intervention will be assessed by the participants’ attendance, their views of PBM, recruitment, and attrition rates. Mixed-effects linear regression modelling will be used to assess the effect of PBM on continuous, outcome measures. We will use the intention-to-treat principle in that we will include everyone in the analysis, whether they complete the treatment or not. We will also conduct sensitivity analysis that only includes those who completed the per-protocol intervention and will report the proportion of participants lost to follow-up.

### 4.1. Recruitment

Participants will be recruited from the IBD and Young Adult Support outpatient clinics at Mater Health from the patient lists of two of the study investigators (TE, JB). The study will employ rolling recruitment until the sample size of 28 participants is reached. Recruitment will be initiated during outpatient appointments, and those who are interested will meet with the research assistant/investigator who will explain the study in more detail, conduct the initial screening, and for those interested in taking part, organise the initial appointment when written informed consent is gained.

### 4.2. Participant Selection

Since this is a study of PBM in young adults with IBD, main eligibility criteria will include participants’ age, confirmed IBD diagnosis and the presence of fatigue. 

Inclusion Criteria:

- Age 18–35 years.
- Diagnosis of IBD made by gastroenterologist.
- Fatigue symptoms (FACIT F score < 30).
- Verbal and written English proficiency that will enable them to participate.
- Ability to provide informed consent.
- Patients of the IBD outpatient clinics at Mater Hospital.
- Individuals who are able to attend the 10 weekly PBM treatment sessions as well as the additional sessions at the baseline and at the end of the study.

Exclusion Criteria:

- Individuals with major psychiatric illness (psychosis, Post-Traumatic Stress Disorder, substance abuse/dependence) as its treatment and symptoms could prevent participation in the programme.
- Individuals without conversational or written English.
- Major surgery booked in the next 6 months as this would preclude participation.
- Pregnant women, as there are no studies to date of PBM in pregnancy.

## 5. PBM Therapy Intervention, Dose Parameters, Application Sites, and Safety

**Figure 1:**
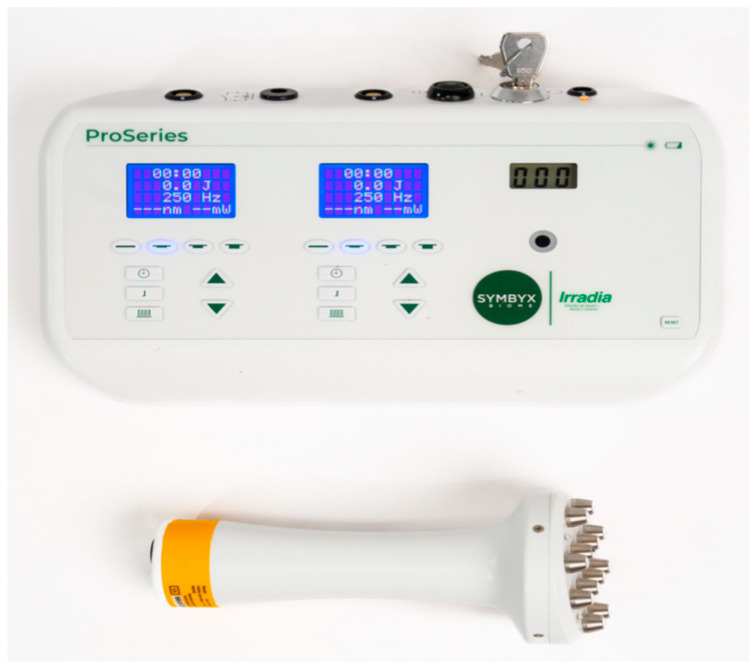
Image of the multiple-diode laser device.

The device to be used is the ProSeries device (SYMBYX Pty Ltd., Sydney, Australia) multi-diode 904 nm array (Figure 1). 

Treatment dose parameters with the PBM device will be applied as shown in Table 1.

**Figure 2:**
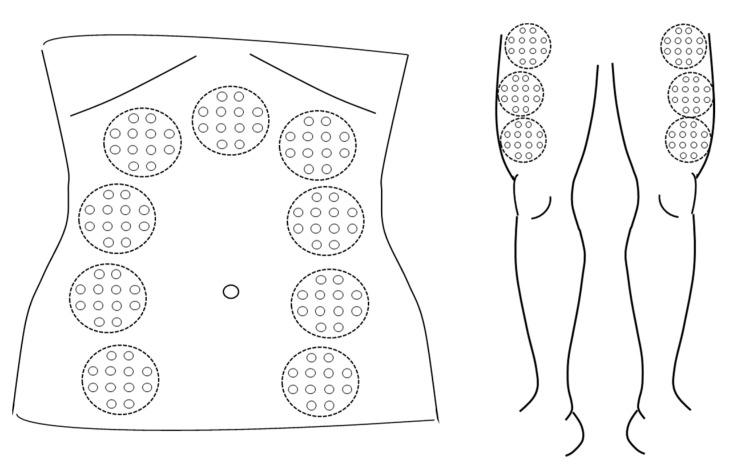
Sites for abdominal and anterior thigh applications of PBM.

A trained operator will administer the PBM. Dosing and application protocols for the study are based on previous studies by one of the authors (E-LL). At each weekly appointment, PBMt application will be on the abdomen (9 sites) and bilateral anterior thigh (6 sites on left and right leg) (Figure 2).

**Figure 3:**
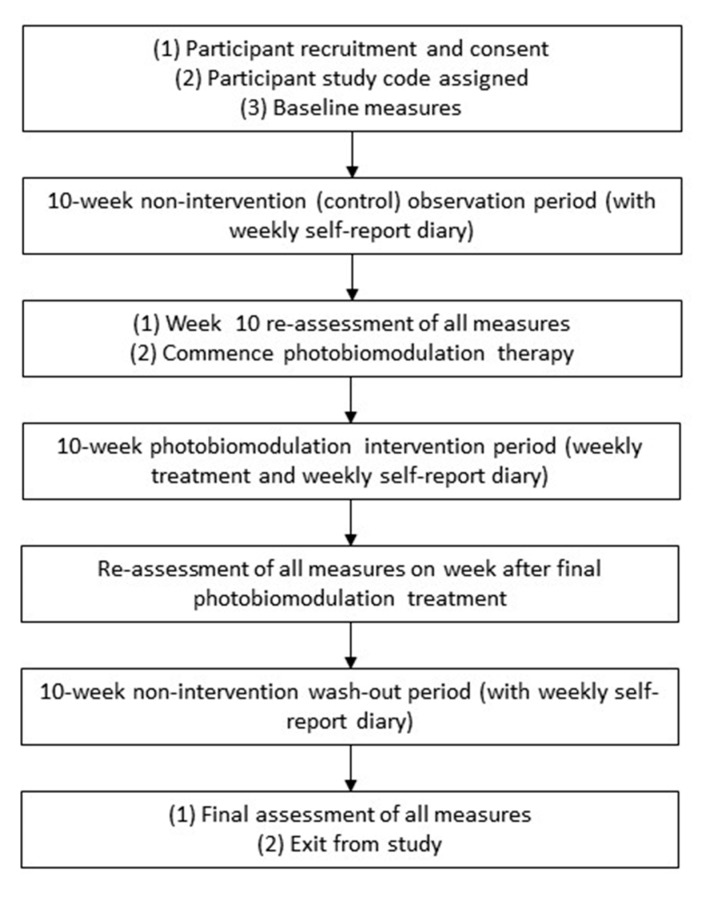
Flow diagram of study.

The flow diagram in Figure 3 illustrates the flow of the participants through the study. 

### PBM Safety

Photobiomodulation therapy (PBMt) is a safe, non-invasive, non-pharmacological method for treating a range of symptoms and signs across a range of conditions. Sensitivity to light has been reported in 2–15% of PBM trials participants, in the form of temporary dizziness, increase in pain, tiredness, or euphoria. Several studies have concluded that there are no long-term adverse effects of PBM in vitro [28], in vivo, including non-human primates [29], and in human models [30,31,32]. We therefore do not expect the participants to experience any adverse events. However, should this occur, the participants will be provided with advice and care by their doctor as required; the event will be recorded as part of the study outcomes; and the institutional human research ethics committee will be informed.

## 6. Study Outcomes and Procedures

Outcomes chosen for the study are valid, reliable, and routinely used in the setting of IBD and chronic illness. 

### 6.1. Study Outcomes

During the first and each subsequent assessment, participants will be seated comfortably, and will complete the following questionnaires:

1. The primary outcome measure will be the Functional Assessment of Chronic Illness Therapy—Fatigue (FACIT-Fatigue) scale, designed to assess fatigue and its impact on daily activities and functioning in chronic diseases over the past week. Thirteen items (taking two minutes to complete) include tiredness, weakness, listlessness, lack of energy, and the impact of these feelings on daily functioning, such as sleeping and social activities [33]. The psychometric properties of the FACIT-Fatigue scale have been established in a range of chronic health conditions, including Parkinson’s disease, kidney dialysis, cancer, stroke, HIV, and iron-deficiency anaemia. In the latter, the scale was found to be stable over time (ICC = 0.87), internally consistent (α = 0.93), and it demonstrated convergence with other conceptually relevant scales such as SF-36 Vitality sub-score (r = 0.74) [33].
2. The DASS is a validated questionnaire that consists of three scales measuring depression, anxiety, and stress [34]. The short version, DASS 21 questionnaire, will be used in the study as its validity is comparable with the long version and it will be easier for the participants to complete.
3. Short Form-36 measures health-related quality of life (36 items taking 7 min to complete). Published reliability statistics for the SF-36 have exceeded the minimum standard of 0.70 recommended for measures used in group comparisons and have exceeded 0.80 in many studies. Reliability estimates for the physical and mental summary scores usually exceed 0.90. Reliability coefficients for the SF-36 scales and summary measures have been replicated across more than 20 patient groups [35].
4. International Physical Activity Questionnaire (IPAQ) is a 7-day recall tool that measures domains of physical activities people do as part of their everyday lives (i.e., activities at work, as part of house and yard work, transport from place to place, and in spare time for recreation, exercise or sport) (27 items taking 8 min if all items require answering). The IPAQ has been validated in many studies [36], which demonstrated that Intraclass correlation coefficients (ICC) were stable between days and the ICC for total activity scores was highest at 0.93 (CI: 0.86 to 0.97). Total activity scores were also significantly related to pedometer step counts (Pearson r = 0.66, *p* < 0.01).
5. Patient-specific Functional Scale (PSFS) quantifies activity limitation and assesses function, allowing patients to nominate any activity they may have difficulty performing due to their health condition. The PSFS takes 3 min to complete. The PSFS has been used extensively and found to be psychometrically and clinimetrically sound in a range of clinical populations with musculoskeletal conditions [37].

Participants will be provided with a ‘sampling kit’ during the first appointment and prior to each subsequent assessment. The sampling kit will contain the pathology request slips for blood (erythrocyte sedimentation rate (ESR) and C-reactive protein (CRP)) and stool samples (faecal calprotectin (FCP)) as part of standard care; and the stool sampling requirements for microbiome analysis by a commercial entity (Microba Life Sciences, Brisbane, Australia). 

Participants will be advised on how to collect stool samples at home prior to the assessment appointment. After study assessments (and/or treatment) have been completed, the participants will be advised to attend pathology for drawing of blood samples, and to deliver the FCP stool sample. Once pathology results become available in the patient record, these will be copied by the research assistant across to the study Excel data sheet in coded form. 

Stool samples for microbiome analysis will be collected by participants at home using the Microba sampling kit, received by the investigator at the relevant appointment, coded and placed securely by the researcher in a −80 °C freezer sample storage in a PC2-compliant laboratory. Samples will be analysed as a ‘job lot’, including DNA extraction, library preparation, shotgun metagenomic sequencing to target depth of 3 Gb, and taxonomic and functional profiling. 

During the first, baseline appointment (taking approximately one hour), consenting participants will be provided with a self-report pain diary (numeric pain rating scale) which they will complete on a weekly basis. After the first appointment, the participant enters a control period of 10 weeks during which no further study-related appointments will be scheduled.

After 10 weeks of no PBM intervention, the pain diary will be gathered from participants and replaced with a new diary to complete in the following 10 weeks during the treatment phase. The same questionnaires and sample collection completed at the first appointment will be repeated at the second assessment appointment. PBM treatment will commence at the second assessment appointment which will take approximately one hour.

The intervention phase consists of 10 PBM treatments, once weekly. Consenting participants will attend at a regular clinic time to receive PBM intervention. Each appointment will take 30 min. At each appointment, participants will be reminded to complete the self-report pain diary, and this will be checked for compliance.

At the end of the treatment phase all questionnaires will be repeated, physical measures will be re-assessed, and self-report pain diaries retrieved from participants. Participants will be asked to complete the post-PBM evaluation survey to establish the acceptability/tolerability of the study requirements and intervention. The survey will contain a mixture of questions including open-ended free text questions regarding PBM acceptability and their views of any changes they experienced as a result of the intervention.

After the post-PBM appointment, participants will enter a second observational (no PBM treatment) period of 10 weeks during which they will continue to complete a weekly study pain diary.

The study will end after 30 weeks, when all questionnaires will be repeated, measures will be re-assessed (final blood and stool samples collected) and self-report diaries retrieved from participants before they exit the study.

### 6.2. Microbiome Analysis

Stool samples for microbiome analysis will be collected from participants on four occasions: at baseline first appointment, after the first 10-week non-intervention period, i.e., pre-PBM, post-PBM, and at the final appointment after the 10-week washout period. Participants will be asked to provide a stool sample using the Microba sampling kit (with instructions) provided to them prior to each of the relevant appointments. 

Microbiome analysis will describe the species and functional profiles analysed using a time dependent analysis to determine the effect of the intervention on alpha and beta diversity on a per patient basis. We will also examine the effect of the intervention on the relative abundance of known beneficial and/or pathogenic microorganisms. 

### 6.3. Safety Considerations

The overall monitoring of the study, including all aspects of safety, research conduct, and data collection and storage, will be conducted by the Mater Misericordiae Ltd. HREC. Researchers will record all adverse and serious adverse events in the trial database, and report them to the Mater HREC, in line with Good Clinical Practice Guidelines.

### 6.4. Data Management and Protection and Statistical Analysis

Completed questionnaires and blood and stool samples will be coded. Data will be stored in a re-identifiable form with linkage of the data to patient details only able to be performed by the study PI and research assistant. Participant data will be identifiable by a unique code given to each participant at the time of enrolment. Unique patient identifiers, names, and contact details will only be accessible by the researchers. All paper-based study information will be kept in an access-limited security swipe room, inside a locked filing cabinet, while electronically collected data will be kept on servers based within the Mater and protected by a firewall and other security measures.

### 6.5. Sample Size Calculation

We have calculated that the sample size required to achieve 80.0% power to reject the null hypothesis, for the effect size of 0.60 and a significance level (alpha) of 0.050, using a two-sided paired t-test, will be 24. From researchers’ experience, the most likely dropout rate in this type of study is between 5–10%. Considering the 5–10% dropout, we will therefore aim to recruit a total sample size of 28.

## 7. Discussion

The study will advance the knowledge about the feasibility and acceptability of PBM in IBD and provide data on the preliminary effectiveness of PBM on fatigue, depression, pain, microbiome diversity, and quality of life in individuals with IBD. The study results will be utilized to determine whether further research is required (based on the potential efficacy of the intervention) and the optimal sample size and design for a future RCT. 

This study is the first to assess PBM intervention in youth with IBD who experience high levels of fatigue, depression, pain and significantly impaired quality of life. Potential benefits of PBM may be particularly relevant in youth with IBD as they could affect their future education, employment, social and overall functioning, and long-term prognosis. 

The study will be using a PBM protocol adapted for individuals with IBD from previous PBM research in other cohorts that, if proven feasible, may become integrated into the treatment pathways for IBD. 

This study has the potential to validate PBM as a feasible intervention for fatigue, depression, and pain in IBD and translate this approach to other chronic diseases. The inclusion of both self-reported psychosocial and physical outcomes may provide additional insights into the relationship between IBD, fatigue, depression, and pain and their responses to PBM.

As this is a pilot, single-arm study, we will not be able to exclude the placebo effect, as any positive results could be due to treatment, placebo, or a combination of the two. To minimise the effect of this, the study will have a 10-week non-intervention period, followed by the 10-week PBM intervention. The 10-week post-intervention period will attempt to elicit whether any ongoing improvements will remain. We anticipate that any positive results would need to be followed up with an RCT with an adequate sample size, updated recruitment strategies, and study procedures.

## Figures and Tables

**Table 1:** Laser PBM device parameters.

Device Parameters	Laser Cluster to Abdomen	Laser Cluster to Anterior Thighs
Number of laser diodes	12	
Wavelength	GaAs, λ = 904 ± 10 nm	
Frequency	Pulsed output (700 Hz)	Pulsed output (700 Hz)
Optical Output	60 mW each diode (total of 720 mW)	
Spot Size	0.5 cm^2^ each spot	
Energy	95.7 J at each site	43.2 J at each site
Treatment time	2 min 13 s at each site (20 min of total treatment time to abdomen)	1 min at each site (6 min of total treatment time to thighs)
Number of irradiation sites	9 (along the anatomical course of the ascending, transverse and descending colon)	6 (3 sites per leg)
Number of irradiation points	108	72
Total energy delivered	861.3 J	129.6 J
Application mode	Probe stationary in skin contact with firm pressure as participant tolerates, and perpendicular to target area	

## Data Availability

Data will be available in de-identified form, from three months after the initial results publication, from the principal investigator and subject to approval by the principal investigator.

## References

[1] Graham D.B., Xavier R.J. (2013). From genetics of inflammatory bowel disease towards mechanistic insights. Trends Immunol..

[2] Larsen S., Bendtzen K., Nielsen O.H. (2010). Extraintestinal manifestations of inflammatory bowel disease: Epidemiology, diagnosis, and management. Ann. Med..

[3] Gracie D.J., Ford A.C. (2016). Psychological Comorbidity and Inflammatory Bowel Disease Activity: Cause or Effect?. Clin. Gastroenterol. Hepatol..

[4] Hindryckx P., Laukens D., D’Amico F., Danese S. (2018). Unmet Needs in IBD: The Case of Fatigue. Clin. Rev. Allergy Immunol..

[5] Saraiva S., Cortez-Pinto J., Barosa R., Castela J., Moleiro J., Rosa I., Da Siva J.P., Pereira A.D. (2019). Evaluation of fatigue in inflammatory bowel disease—A useful tool in daily practice. Scand. J. Gastroenterol..

[6] Jason L.A., Evans M., Brown M., Porter N. (2010). What is Fatigue? Pathological and Nonpathological Fatigue. Am. Acad. Phys. Med. Rehabil..

[7] Martin-Subero M., Anderson G., Kanchanatawan B., Berk M., Maes M. (2016). Comorbidity between depression and inflammatory bowel disease explained by immune-inflammatory, oxidative, and nitrosative stress; tryptophan catabolite; and gut-brain pathways. CNS Spectr..

[8] Mikocka-Walus A., Pittet V., Rossel J.-B., Von Känel R. (2016). Symptoms of Depression and Anxiety Are Independently Associated With Clinical Recurrence of Inflammatory Bowel Disease. Clin. Gastroenterol. Hepatol..

[9] Villoria A., García V., Dosal A., Moreno L., Montserrat A., Figuerola A., Horta D., Calvet X., Lázaro M.J.R. (2017). Fatigue in out-patients with inflammatory bowel disease: Prevalence and predictive factors. PLoS ONE.

[10] Regueiro M., Greer J.B., Szigethy E. (2017). Etiology and Treatment of Pain and Psychosocial Issues in Patients With Inflammatory Bowel Diseases. Gastroenterology.

[11] Perler B.K., Ungaro R., Baird G., Mallette M., Bright R., Shah S., Shapiro J., Sands B.E. (2019). Presenting symptoms in inflammatory bowel disease: Descriptive analysis of a community-based inception cohort. BMC Gastroenterol..

[12] Nampo F.K., Cavalheri V., Ramos S.P., Camargo E.A. (2016). Effect of low-level phototherapy on delayed onset muscle soreness: A systematic review and meta-analysis. Lasers Med. Sci..

[13] Vanin A.A., Verhagen E., Barboza S.D., Costa L.O.P., Leal-Junior E.C.P. (2018). Photobiomodulation therapy for the improvement of muscular performance and reduction of muscular fatigue associated with exercise in healthy people: A systematic review and meta-analysis. Lasers Med. Sci..

[14] Miranda E.F., de Oliveira L.V.F., Antonialli F.C., Vanin A.A., de Carvalho P.T.C., Leal-Junior E.C.P. (2015). Phototherapy with combination of super-pulsed laser and light-emitting diodes is beneficial in improvement of muscular performance (strength and muscular endurance), dyspnea, and fatigue sensation in patients with chronic obstructive pulmonary disease. Lasers Med. Sci..

[15] Ferraresi C., Huang Y.-Y., Hamblin M.R. (2016). Photobiomodulation in human muscle tissue: An advantage in sports performance?. J. Biophotonics.

[16] Oliveira M.X., Toma R.L., Jones B.J.L., Cyprien T.P., Tier M.R., Wallace C.A., Renno A.C.M., Sabapathy S., Laakso E.-L., Hamblin M.R., Carroll J.D., Arany P. (2017). Effects of photobiomodulation therapy (pulsed LASER 904 nm) on muscle oxygenation and performance in exercise-induced skeletal muscle fatigue in young women: A pilot study. Mechanisms of Photobiomodulation Therapy XII.

[17] Cassano P., Petrie S.R., Mischoulon D., Cusin C., Katnani H., Yeung A., Taboada L.D., Archibald A., Bui E., Baer L. (2018). Transcranial Photobiomodulation for the Treatment of Major Depressive Disorder. The ELATED-2 Pilot Trial. Photomed. Laser Surg..

[18] Caldieraro M.A., Cassano P. (2019). Transcranial and systemic photobiomodulation for major depressive disorder: A systematic review of efficacy, tolerability and biological mechanisms. J. Affect. Disord..

[19] Videnovic A., Klerman E.B., Wang W., Marconi A., Kuhta T., Zee P.C. (2017). Timed Light Therapy for Sleep and Daytime Sleepiness Associated With Parkinson Disease: A Randomized Clinical Trial. JAMA Neurol..

[20] Chow R.T., Johnson M.I., Lopes-Martins R.A.B., Bjordal J.M. (2009). Efficacy of low-level laser therapy in the management of neck pain: A systematic review and meta-analysis of randomised placebo or active-treatment controlled trials. Lancet.

[21] Chow R.T., Armati P.J. (2016). Photobiomodulation: Implications for Anesthesia and Pain Relief. Photomed. Laser Surg..

[22] Clijsen R., Brunner A., Barbero M., Clarys P., Taeymans J. (2017). Effects of low-level laser therapy on pain in patients with musculoskeletal disorders: A systematic review and meta-analysis. Eur. J. Phys. Rehabil. Med..

[23] Ezzati K., Laakso E.-L., Fekrazad R., Hasannejad A., Salari A. (2020). The beneficial effects of high-intensity laser therapy and adding co-interventions to it on musculoskeletal pain management: A systematic review. Lasers Med. Sci..

[24] Liebert A., Bicknell B., Johnstone D.M., Gordon L.C., Kiat H., Hamblin M.R. (2019). ‘‘Photobiomics’’: Can Light, Including Photobiomodulation, Alter the Microbiome?. Photobiomodulation Photomed. Laser Surg..

[25] Zanotta N., Ottaviani G., Campisciano G., Poropat A., Bovenzi M., Rupel K., Gobbo M., Comar M., Di Lenarda R., Biasotto M. (2020). Photobiomodulation modulates inflammation and oral microbiome: A pilot study. Biomarkers.

[26] Laakso E.L., Ewais T. (2023). A Holistic Perspective on How Photobiomodulation May Influence Fatigue, Pain, and Depression in Inflammatory Bowel Disease: Beyond Molecular Mechanisms. Biomedicines.

[27] Tinsley A., Macklin E.A., Korzenik J.R., Sands B.E. (2011). Validation of the Functional Assessment of Chronic Illness Therapy-Fatigue (FACITF) in patients with inflammatory bowel disease. Aliment. Pharmacol. Ther..

[28] Khan I., Tang E., Arany P. (2015). Molecular pathway of near-infrared laser phototoxicity involves ATF-4 orchestrated ER stress. Sci. Rep..

[29] Moro C., Torres N., Arvanitakis K., Cullen K., Chabrol C., Agay D., Darlot F., Benabid A.L., Benabid J. (2017). No evidence for toxicity after long-term photobiomodulation in normal non-human primates. Exp. Brain Res..

[30] Samoilova K.A., Zimin A.A., Buinyakova A.I., Makela A.M., Zhevago N.A. (2015). Regulatory systemic effect of postsurgical polychromatic light (480–3400nm) irradiation of breast cancer patients on the proliferation of tumor and normal cells in vitro. Photomed. Laser Surg..

[31] Moskvin S.V., Khadartsev A.A. (2016). Laser Light—Can It Be Harmful?. Technologies.

[32] Liebert A., Bicknell B., Laakso E.-L., Tilley S., Jalilitabaei P., Kiat H., Mitrofanis J. (2022). Remote photobiomodulation treatment for the clinical signs of Parkinson’s disease: A case series conducted during COVID-19. Photobiomodulation Photomed. Laser Surg..

[33] Acaster S., Dickerhoof R., DeBusk K., Bernard K., Strauss W., Allen L.F. (2015). Qualitative and quantitative validation of the FACIT-fatigue scale in iron deficiency anemia. Health Qual. Life Outcomes.

[34] Lovibond P.F., Lovibond S.H. (1995). The structure of negative emotional states: Comparison of the Depression Anxiety Stress Scales (DASS) with the Beck Depression and Anxiety Inventories. Behav. Res. Ther..

[35] Ware J.E., Gandek B. (1998). Overview of the SF-36 Health Survey and the International Quality of Life Assessment (IQOLA) Project. J. Clin. Epidemiol..

[36] Gauthier A.P., Lariviere M., Young N. (2009). Psychometric Properties of the IPAQ: A Validation Study in a Sample of Northern Franco-Ontarians. J. Phys. Act. Health.

[37] Horn K.K., Jennings S., Richardson G., Van Vliet D., Hefford C., Haxby Abbott J. (2012). The Patient-Specific Functional Scale: Psychometrics, Clinimetrics, and Application as a Clinical Outcome Measure. Orthop. Sports Phys. Ther..

